# The Assessment of Agrobiological and Disease Resistance Traits of Grapevine Hybrid Populations (*Vitis vinifera* L. × *Muscadinia rotundifolia* Michx.) in the Climatic Conditions of Crimea

**DOI:** 10.3390/plants10061215

**Published:** 2021-06-15

**Authors:** Vladimir Volynkin, Irina Vasylyk, Vitalii Volodin, Elizaveta Grigoreva, Dmitry Karzhaev, Ekaterina Lushchay, Pavel Ulianich, Vladimir Volkov, Valentina Risovannaya, Sofiya Blinova, Jakov Alekseev, Svetlana Gorislavets, Vladimir Likhovskoi, Aleksandar Beatovic, Elena Potokina

**Affiliations:** 1 All-Russian National Research Institute of Viticulture and Winemaking ‘Magarach’ RAS, 298600 Yalta, Russia; volynkin@ukr.net (V.V.); kalimera@inbox.ru (I.V.); mgr.magarach@gmail.com (V.V.); L.Grigoreva@gmail.com (E.G.); karzhaevd@gmail.com (D.K.); biogen@magarach-institut.ru (E.L.); p.ulianich@gmail.com (P.U.); vol-j@mail.ru (V.V.); vrisovan@rambler.ru (V.R.); jalex@syntol.ru (J.A.); goricvet_2@rambler.ru (S.G.); lihovskoy@gmail.com (V.L.); 2 Institute of Forest and Natural Resources Management, Saint Petersburg State Forest Technical University, 194021 St. Petersburg, Russia; 3 Information Technologies and Programming Faculty, ITMO University, 197101 St. Petersburg, Russia; abeatovic@itmo.ru; 4 All-Russian Research Institute of Agricultural Microbiology, 196608 St. Petersburg, Russia; 5 Syntol, 127434 Moscow, Russia; sofya.blinova@yandex.ru; 6 All-Russian Research Institute of Agricultural Biotechnology, 127434 Moscow, Russia

**Keywords:** grapevine, the Crimea, abiotic and biotic stress factors, agrobiological traits, disease resistance loci, *Muscadinia rotundifolia*, hybrids, RADseq

## Abstract

The Crimean autochthonous grape varieties are unique by their origin and serve as a valuable source for breeding new cultivars with increased salt and frost resistance, as well as high-quality berries. However, they suffer from fungal pathogens, as the dry and hot summer months contribute to the epiphytotic course of diseases. An increase in the resistance of Crimean grape varieties is currently achieved through interspecific hybridization. In this study, we describe the genetic and agrobiological diversity of three hybrid populations obtained using the *Vitis* interspecific hybrid ‘Magarach 31-77-10′ as a female parent and *Muscadinia rotundifolia* × *Vitis vinifera* BC5 hybrid plants as male parents. The hybrid nature of the populations was assessed using RADseq high-throughput genotyping. We discovered 12,734 SNPs, which were common to all three hybrid populations. We also proved with the SSR markers that the strong powdery and downy mildew resistance of the paternal genotypes is determined by the dominant *Run1/Rpv1* locus inherited from *M. rotundifolia*. As a result, the disease development score (R, %) for both mildew diseases in the female parent ‘Magarach 31-77-10’ was three times higher than in male parents 2000-305-143 and 2000-305-163 over two years of phytopathological assessment. The highest values of yield-contributing traits (average bunch weight ~197 g and 1.3 kg as yield per plant) were detected in the population 4-11 (♀M. No. 31-77-10 × 2000-305-163). Despite the epiphytotic development of PM, the spread of oidium to the vegetative organs of hybrids 4-11 did not exceed 20%. Some hybrid genotypes with high productivity and resistance to pathogens were selected for further assessment as promising candidates for new varieties.

## 1. Introduction

The Crimean Peninsula belongs to the northern part of the distribution area of *Vitis vinifera* L. subsp. *sylvestris* (Gmel.) Hegi, which is considered the wild forest ancestor of cultivated grapes. Viticulture on the Crimean Peninsula has a long and ancient history; there is a hypothesis that the wild grape, preserved here from the Tertiary period, was collected for food by local tribes (Taurians) and then bred for cultivation [1]. In Crimea, populations of *V. vinifera* L. subsp. *sylvestris* still occur in natural forest habitats and are of particular value for breeding due to their high salt and frost resistance, quality of berries, productivity, and ecological plasticity. Numerous local varieties cultivated in ancient times in the Crimea are assumed to derive from the wild-growing grapevine. However, the Crimea was a region with frequent changes of native populations, cultures, and religions in the past. Accordingly, viticulture in the peninsula underwent periods of prosperity and decline along with the introduction of new germplasm of grapes. A recent ampelographic study of 80 local Crimean grapevine varieties from the collection of the Institute of Vine and Wine Magarach in Yalta (Crimea) reported that about 45% of local Crimean varieties belong to the Eastern European eco-geographical group (*V. vinifera* subsp. *sativa* convar *orientalis* Negr.), 38% to the eco-geographical group of the Black Sea Basin (*V. vinifera* subsp. *sativa* convar *pontica* Negr.), and 17% to the Western European group (*V. vinifera* subsp. *sativa* convar *occidentalis* Negr.) [2].

Although the local grape varieties of Crimea are distinguished by their unique wine qualities, drought resistance, and tolerance to high temperatures, they have some disadvantages. Most of the Crimean local varieties have functionally female flowers. This significantly affects the stability of fertilization, so the yield directly depends on favorable weather conditions [3]. Additionally, varieties with functionally female flowers usually show lower productivity that varies between 222 and 289 kg ha^−1^, while the few Crimean autochthonous varieties with hermaphrodite flowers demonstrate a yield up to 622 kg ha^−1^ [4]. Increasing the productivity of Crimean native varieties is mainly carried out by clone selection [5].

Another weakness of the Crimean autochthonous varieties is low resistance to pathogens, especially to oidium (*Erysiphe necator*), since the epiphytotic course of this disease is facilitated by dry and hot summer months. The importance of breeding resistant grape varieties to be cultivated in the Crimea is highlighted by the fact that in practice, plant protection treatments often encounter the phenomenon of insufficient efficiency, even when using the most effective fungicides [6]. Experiments with crosses between the local Crimean varieties from different eco-geographical groups yielded offspring with higher productivity, a shorter vegetation cycle, and earlier ripening. However, it is hardly possible to create varieties with complex resistance to fungal pathogens and phylloxera using only intraspecific crosses within *V. vinifera* [7]. Thus, an increase in the adaptive capacity of Crimean varieties is currently achieved through interspecific hybridization.

Forty-three cross combinations were analyzed in the Research Institute of Viticulture and Winemaking ‘Magarach’ in Yalta in 2008–2016 involving both the Crimean native varieties and intraspecific hybrids resistant to diseases (e.g., Seyve Villard, Joannes Seyve, and Seibel). As a result, new Crimean grape cultivars (e.g., Antei Magarachskii, Tsitronnyi Magaracha, Podarok Magaracha, Pervenets Magaracha, and Spartanets Magaracha) were released having higher disease resistance and improved quality [8]. During the crossing experiments, several elite hybrid genotypes were identified with the highest breeding value when selecting for resistance to oidium. Among them was the genotype Magarach No. 31-77-10, which was successfully used as a maternal parent in most performed crosses.

The North American species *V. rotundifola* Michx (syn. *Muscadinia rotundifolia*) was reported as the most promising source of grapevine resistance to downy mildew (*Plasmopara viticola*) and powdery mildew (*Erysiphe necator*), which demonstrates almost complete immunity to fungal pathogens, while introgression from other North American species provided only partial resistance [9,10]. The main issue with the successful interspecies crosses between *V. vinifera* and *M. rotundifola* is that they belong to two different sub-genera (*Euvitis* and *Muscadiana* Planch.) and have a different diploid set of chromosomes (2n = 2x = 38 and 2n = 2x = 40, respectively). However, as a result of a few successful attempts, in 1919, some partly fertile interspecific hybrids were obtained [11]. One of them, the famous hybrid NC6-15, was used as the resistant parent in a series of pseudo-backcrosses with *V. vinifera* varieties [12]. The purpose of the performed backcrosses was to saturate the genome of interspecific hybrids with the genes of cultivated grapes, but with the preservation of the resistance loci inherited from *Muscadinia rotundifolia*. To this end, at each backcross step, a resistant individual was crossed with a different susceptible *V. vinifera* genotype to prevent inbreeding depression. As a result, BC5 and BC6 populations were obtained, in which resistance to powdery and downy mildew segregated in a 1:1 ratio [12,13]. The BC5 progeny Mtp 3294 (VRH 3082-1-42 × *V. vinifera cv.* Cabernet Sauvignon) was employed for the fine mapping, positional cloning, and functional characterization of the unique resistance locus from *M. rotundifolia*, containing two closely related genes *MrRUN1* and *MrRPV1*, which confer strong resistance to powdery mildew and downy mildew, respectively [14].

The half-siblings of Mtp 3294, the genotypes 2000-305-143 and 2000-305-163, were obtained from crossing the same female parent VHR 3082-1-42 and *V. vinifera cv*. Regent. These genotypes were kindly provided by Prof. R. Eibach (Institut für Rebenzüchtung Geilweilerhof, Germany) to the Research Institute of Viticulture and Winemaking ‘Magarach’ in Yalta (Crimea). In 2011, the 2000-305-143 and 2000-305-163 genotypes were involved in the local breeding program of the ‘Magarach’ Institute, serving as donors of loci of resistance in the crosses with the elite parental female Magarach No. 31-77-10, which showed the highest breeding value when selecting for resistance to oidium in all crosses carried out earlier.

In this article, for the first time, we genetically assessed the obtained hybrid populations using SNP markers generated by restriction-site-associated DNA sequencing (RADseq) [15]. Discovering thousands of SNP loci, RADseq has become a valuable method in population studies, outperforming other molecular marker techniques (e.g., microsatellites) in applications that require individual-level genotype information, such as quantifying relatedness and individual-level heterozygosity [16]. Previously, we successfully employed the ddRADseq method for genotyping of non-model species [17,18] and found the technique as an efficient and low-cost approach to de novo SNP discovery.

The aim of our study was the assessment of the agrobiological and disease resistance traits of hybrid populations of grapevine, which presumably carry the introgressions of the species *M. rotundifolia*, in ecological conditions of the Crimea, as a specific area of viticulture.

## 2. Results

### 2.1. Pedigree of Grapevine Hybrid Populations with Introgressions from M. rotundifolia Developed in the Research Institute of Viticulture and Winemaking ‘Magarach’ (Crimea)

**Figure 1:**
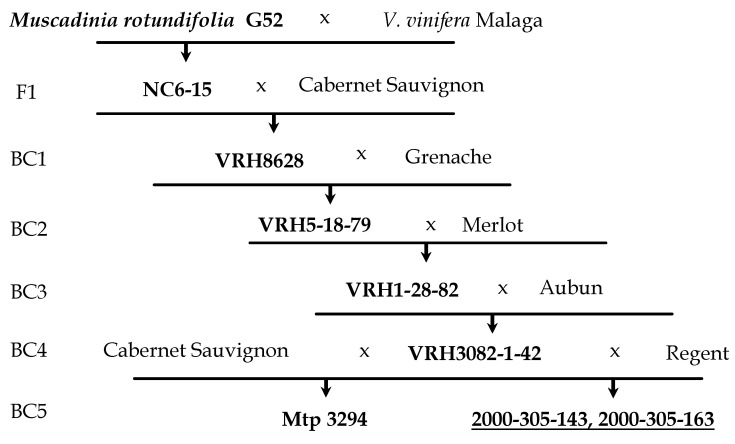
Scheme of backcrosses in order to complete the introgression of genes for resistance to powdery and downy mildew from *M. rotundifolia* to the genetic background of *V. vinifera*. Resistant BC progeny are marked in bold.

**Figure 2:**
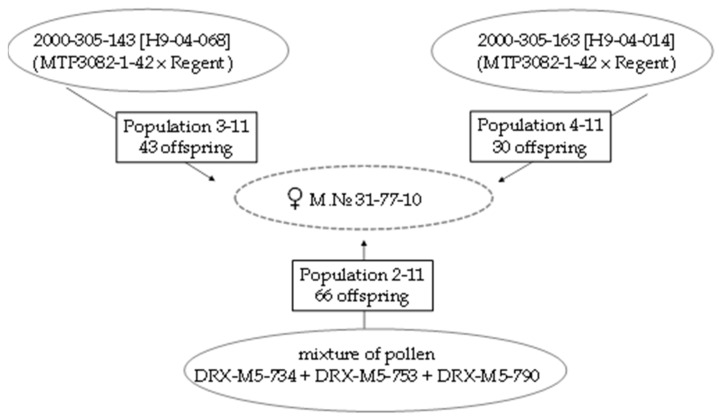
Grapevine hybrid populations (3-11, 4-11, and 2-11) carrying introgressions from *M. rotundifolia* developed in the Research Institute of Viticulture and Winemaking ‘Magarach’ (Crimea) using male parents 2000-305-143 and 2000-305-163 and pollen of DRX-M5 hybrids (respectively) as donors of resistance loci. In all three crosses, the same female parent, Magarach No. 31-77-10 (M. No. 31-77-10), was employed.

The genotype Magarach No. 31-77-10 (‘Nimrang’ × ‘Seibel 13666′) was employed as a female parent in all the crosses performed. Paternal genotypes 2000-305-143 and 2000-305-163 are the BC5 progeny of pseudo-backcrosses, tracing back to the first fertile interspecies (*Vitis vinifera* × *Muscadinia rotundifolia*) hybrid NC6-15 (Figure 1). Both resistant paternal genotypes are expected to carry *MrRUN1* and *MrRPV1* genes from *M. rotundifolia*, since their half-sibling Mtp 3294 was used to dissect those resistance loci [14]. Additionally, 2000-305-143 and 2000-305-163 may also inherit some additional resistance loci from their parent *cv.* Regent—a cultivar with quantitative resistance against downy and powdery mildew released in Germany in 1996 [19]. For *cv*. Regent, at least two resistance loci for *Erysiphe necator* (*Ren3*, *Ren 9*) [20,21] and one resistance locus for *Plasmopara viticola* (*Rpv 3.1*) [22] have been reported. Forty-three progenies were obtained from the cross ♀M. No. 31-77-10 × 2000-305-143 and 30 progenies from the cross ♀M. No. 31-77-10 × 2000-305-163 in 2011 (Figure 2).

Additionally, the maternal plant ♀M. No. 31-77-10 was artificially pollinated by a mixture of pollen collected from DRX-M5 interspecies hybrids. The latter were developed at the Institute of Viticulture and Winemaking ‘Vierul’ (Moldova) as F5 progeny from the interspecific crosses of the famous DRX-55 hybrid (F2 from N.C.6-15 open pollinated) [23]. In 2011, pollen of three hybrids DRX-M5-734, DRX-M5-753, and DRX-M5-790 were sent to the ‘Magarach’ Institute and used to pollinate the female parent Magarach No. 31-77-10, resulting in 66 progenies (Figure 2).

### 2.2. Assessment of the Hybrid Population Relationship Using Genotyping by Sequencing with IlluminaHiSeq2500

To assess the genetic identity and confirm the hybrid nature of the progenies obtained from three crosses, 139 progeny genotypes (66 + 43 + 30) and parental genotypes (female M. No. 31-77-10; male 2000-305-143, 2000-305-163) were genotyped using the double-digest ddRADseq approach [15]. The precise description of the procedure performed has been published previously [17,18]. We constructed 151 ddRAD libraries from the 139 progenies of three hybrid populations and three parents (each in 4 replicates). Sequencing was performed on Illumina HiSeq2500 with single end reads of 150 base pairs.

In total, 309,762,340 high-quality reads were obtained. For the female parent, which was common for all of the three populations, 6,717,571 reads were generated; for male parents 2000-305-143 and 2000-305-163, 4,414,422 and 9,010,505 reads were obtained, respectively. The progeny of population 2-11 provided 140,445,297 reads, the progeny of population 3-11 provided 97,219,515 reads, and the progeny of population 4-11 provided 51,955,030 reads.

Next, the reads were aligned to the *V. vinifera* 12× genome assembly (https://www.ncbi.nlm.nih.gov/assembly/GCF_000003745.3/, accessed on 18 September 2017). For population 2-11, 86.6% of the reads were successfully mapped to the reference genome; for populations 3-11 and 4-11, the percentage of successfully aligned reads was 87.4% and 86.1%, respectively.

Aligned reads were subjected to the SNP calling procedure using the Tassel v.5.2.40 GBSv2 plug-in [24] and Stacks v. 2.53 bioinformatic software [25]. Next, the filtration of raw SNPs using VCFtools was applied to determine high-quality SNPs. As shown in Table 1, Tassel provided a higher number of raw SNPs, while Stacks kept a higher number of filtered SNPs; therefore, subsequent analysis was performed on the Stacks-filtered SNP data set.

**Figure 3:**
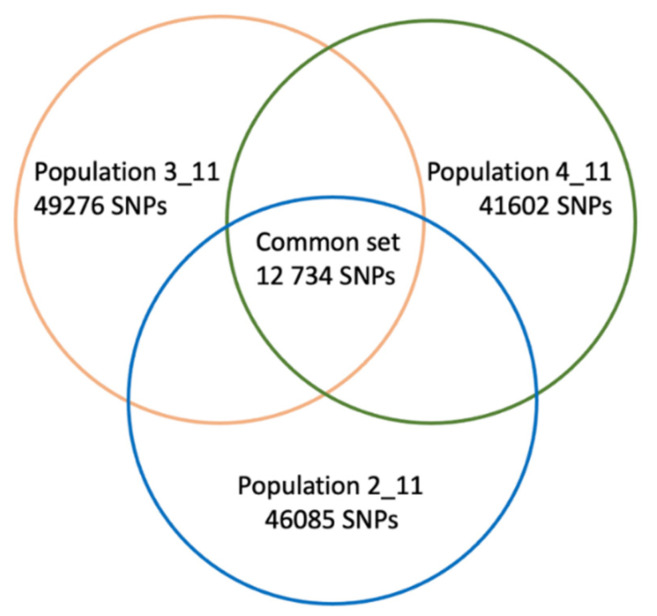
A Venn diagram showing overlapping SNP sets identified when aligning Illumina reads of the hybrid populations 2-11, 3-11, and 4-11 to *V. vinifera* 12× genome assembly using Stacks.

According to the Venn diagram in Figure 3, the three hybrid populations shared a common set of 12,734 SNPs, which were further employed for principal component analysis (PCA). The SNP data set is available in Supplementary Materials, Table S1.

About 17% of SNP missing data were detected across all 152 plants genotyped. The highest number of heterozygous SNP markers were discovered in the female genotype M. No. 31-77-10 (85%), while the corresponding percentage for male 2000-305-143 and 2000-305-163 genotypes was 53% and 43%, respectively (Supplementary Materials, Figure S1 and Table S2).

**Figure 4:**
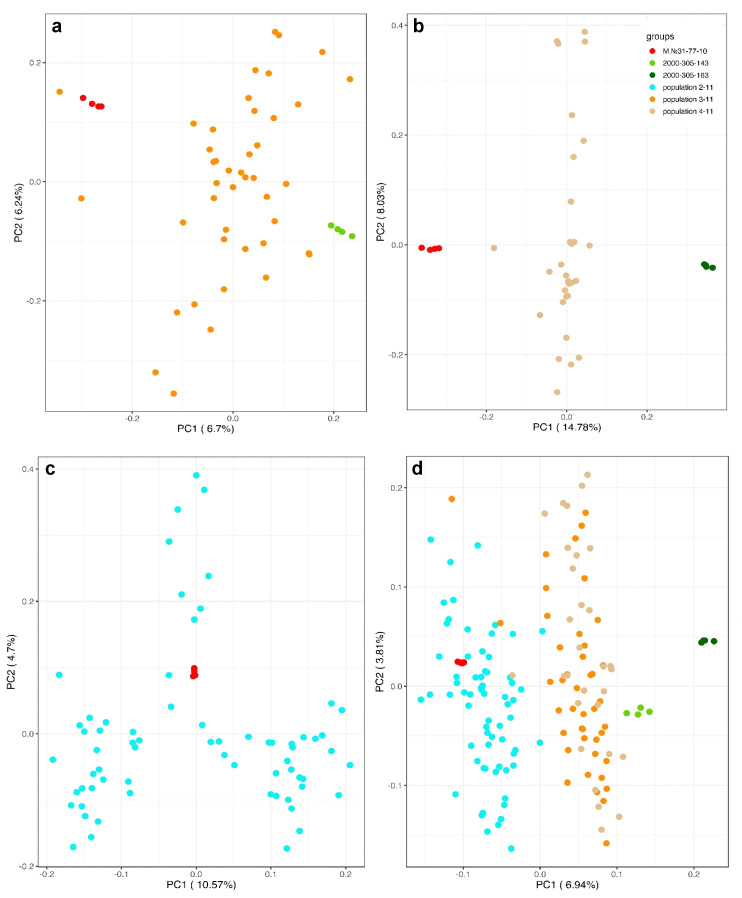
Principal component analysis of the genetic diversity of hybrid populations 3-11 (**a**), 4-11 (**b**), 2-11 (**c**), and all three populations together (**d**) based on a common data set consisting of 12,734 SNPs. All three hybrid populations shared the same maternal genotype, M. No. 31-77-10, marked in black.

First, we examined whether the hybrids of the populations 3-11 and 4-11, for which both female and male parents were determined, show their intermediate genetic background when compared to the maternal and paternal genotypes (Figure 4a,b). Population 4-11, deriving from the cross of female M. No. 31-77-10 and male 2000-305-163, appears quite homogeneous since the progeny are genetically equidistant from both parents, as expected. A substantial variation among the progeny in population 3-11 was observed, though all the progeny were placed on the PCA plot between the parental genotypes (M. No. 31-77-10 and 2000-305-143).

Remarkably, the progeny of population 2-11, obtained as a result of pollination of the maternal genotype M.No.31-77-10 with a mixture of pollen collected from hybrids DRX-M5-734, DRX-M5-753, and DRX-M5-790, split into three non-overlapping clusters on the PCA plot (Figure 4c).

The PCA plot for all three populations taken together shows that populations 3-11 and 4-11 are closely related, as expected, and are genetically separated from population 2-11 (Figure 4d).

### 2.3. Assessment of Variability of Agrobiological Traits among Hybrid Populations

#### 2.3.1. Transit to Generative Stage

All crosses to obtain hybrid populations 2-11, 3-11, and 4-11 were carried out in 2011. In 2012, the seeds were germinated, providing seedlings for the first year of life. In 2013, the seedlings were planted as self-rooted plants in the experimental field. Thus, the hybrid populations have been monitored since 2013.

**Figure 5:**
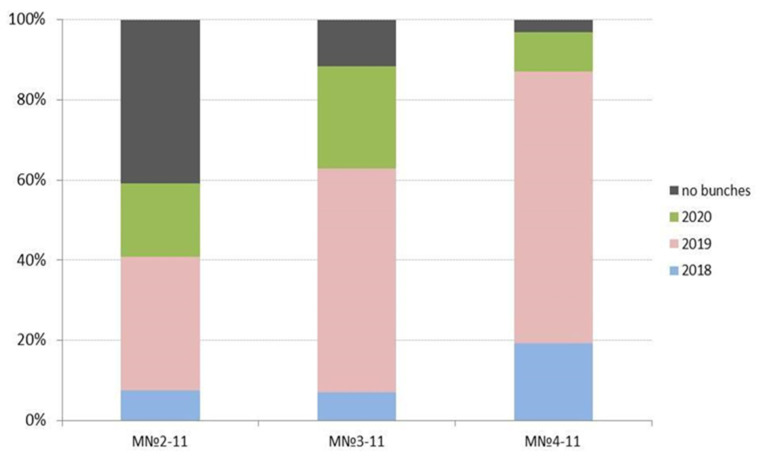
Difference between populations of hybrids 2-11, 3-11, and 4-11, according to the year when the first bunches were recorded.

Long-term observations showed that the seedlings from the three crosses varied greatly in many traits, but most significantly by the year of life, when the first bunches were recorded. As shown in Figure 5, about 40% of the progeny of population 2-11 still did not enter fruiting by 2020, while the progeny from populations 3-11 and 4-11 were more than 90% fertile in 2020. We assume that the remarkable difference can be explained by the complex genetic origin of the 2-11 progeny. In our practice, even in intraspecific crosses, up to 30% of seedlings do not begin to flower, and this is influenced by a number of factors, both genetic and environmental.

#### 2.3.2. Productivity Trait Variation

**Figure 6:**
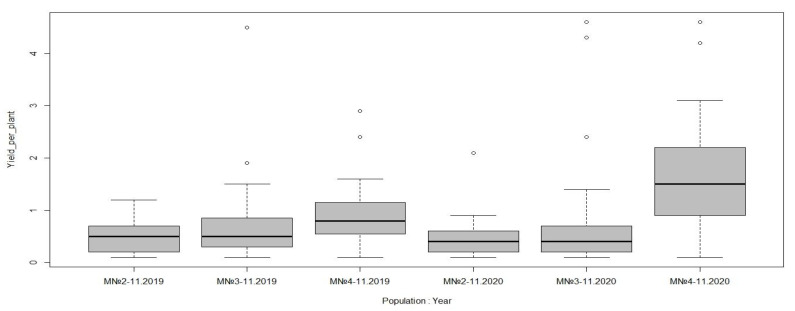
Boxplot of yield per plant (kg) variation between hybrid populations recorded in 2019 and 2020. Boxes indicate the interquartile range. The median for the respective data set is indicated by a horizontal line in the boxplot.

Focusing only on the plants that were flowering by 2019 in each hybrid population, we compared the productivity-related traits between the populations based on the field evaluation data collected in 2019–2020. As shown in Table 2, all three populations were comparable in terms of the number of latent buds, slightly varied by the number of developed shoots, but significantly differed in the number of fertile shoots, the number of inflorescences, and, correspondingly, the number of bunches per plant. The highest values of these yield-contributing traits were shown by population 4-11 (Table 2). As a result, for the progeny of the hybrid population 4-11, the maximum yield per plant was recorded both in 2019 and in 2020 (Figure 6).

### 2.4. Evaluation of the Resistance of the Hybrid Populations to Powdery Mildew (Erysiphe necator) and Downy Mildew (Plasmopara viticola)

#### 2.4.1. Field Assessment of Resistance to Powdery Mildew (PM) and Downy Mildew (DM)

Phytopathological assessment of hybrid populations was performed in 2017, 2018, and 2019 consecutively, under natural conditions in the field, without the use of fungicides. Here, we present the results of phytopathological analyses for 2017 and 2019, since 2018 was unusual in its climatic conditions, which resulted in diseases developing moderately across the entire sample. In general, the resistance score of all three populations to both PM and DM pathogens varied from 5 to 9 according to the OIV-455 and OIV-452 resistance scale [26], meaning intermediate or high resistance. To expose more clearly the interpopulation diversity in terms of resistance to fungal diseases, we also recorded for each population the disease development score (R%) reflecting the percentage of affected leaves per plant and the degree of disease development (see Section 4, “Materials and Methods”).

**Figure 7:**
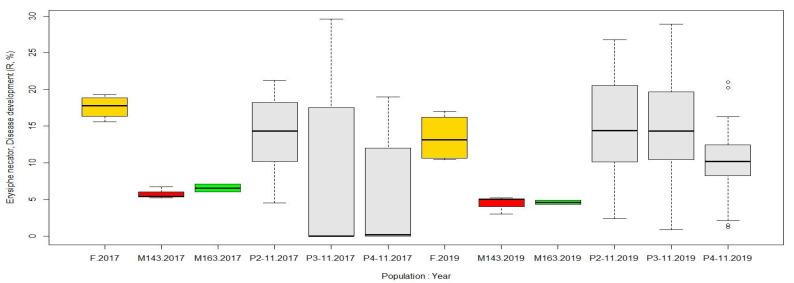
Resistance to powdery mildew (assessed as disease development score (R, %) per plant) of three hybrid populations (colored in light gray) and their parental genotypes (female M. No. 31-77-10, in yellow; male 2000-305-143, in red; male 2000-305-163, in green) under field natural conditions recorded in 2017 and 2019.

**Figure 8:**
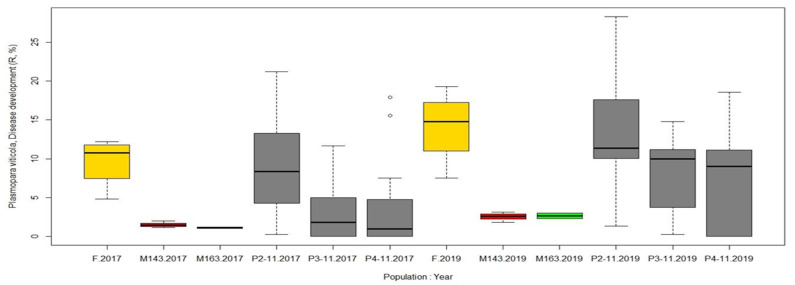
Resistance to downy mildew (assessed as disease development (R, %) per plant) of three hybrid populations (colored in dark gray) and their parental genotypes (female M. No. 31-77-10, in yellow; male 2000-305-143, in red; male 2000-305-163, in green) under field natural conditions recorded in 2017 and 2019.

In this regard, there was a significant difference between the parental genotypes for resistance to both downy and powdery mildew: the percentage of leaves affected by the pathogens in the female parent M. No. 31-77-10 was more than three times higher than in the male parents 2000-305-143 and 2000-305-163 in both 2017 and 2019 (Figure 7 and Figure 8).

The progeny of population 2-11 showed the same level of resistance to both pathogens as their female parent, as confirmed by the unpaired two-sample Wilcoxon test (*p* > 0.05). Their half siblings, though, populations 3-11 and 4-11, were closer to their male parents—genotypes 2000-305-143 and 2000-305-163—in their DM resistance (Figure 8). The percentage of leaves per plant with PM symptoms in populations 3-11 and 4-11 varied significantly depending on the year (Figure 7).

We noticed that in individual genotypes, resistance to each specific pathogen can be expressed to varying degrees. There were genotypes with high resistance to PM but medium resistance to DM, and vice versa. There were also some outstanding genotypes with high resistance to both mildew diseases.

#### 2.4.2. Evaluation of Parental Genotypes with SSR Markers Linked to PM and DM Resistance Loci

The strong powdery mildew resistance of the parental genotypes 2000-305-143 and 2000-305-163 is presumably supported by a single dominant locus Resistance to *Uncinula necator* (*Run1*) inherited from *M. rotundifolia*. To confirm the presence of resistant alleles at this locus in the paternal genotypes, we employed the closest flanking SSR markers VMC4f3.1 and VMC8g9 reported for the *Run1* locus [27] (Table 3). For the VMC8g9 marker, which completely co-segregates with *Run1,* two alleles were registered; among them a fragment of 159 bp has been described as a resistant allele, tracing back to the NC6-15 hybrid [28]. For the second flanking marker, VMC4f3.1, localized at a distance of 0.6 cM from the *Run1* locus, just one allele size of 192 bp was recorded, suggesting the homozygous state of the marker for both paternal genotypes 2000-305-143 and 2000-305-163. The 192 bp allele has also been described as being associated with powdery mildew resistance [28]. 

The chromosomal interval between SSR markers VMC4f3.1 and VMC8g9, flanking *Run1*, is located in linkage group 12 of the *V. vinifera* consensus map [29]. The locus of resistance to downy mildew (Resistance to *Plasmopara viticola* (*RPV1*)) is tightly linked to the *Run1* locus in *M. rotundifolia* × *V. vinifera* BC2 hybrid plants [30]. Merdinoglu et al. [30] were able to link the *Rpv1* locus to the SSR marker VMC1g3.2, while Katula-Debreceni et al. suggested 122 bp as an *Rpv1*-specific allele size [31]. However, Prazzoli et al. [32] signified 118 bp as the allele size linked to resistance at the *Rpv1* locus. In our experiments, we documented the presence of a resistant allele of 118 bp in both paternal genotypes 2000-305-143 and 2000-305-163 but not in the susceptible female parent M. No. 31-77-10 (Table 3).

For a deeper assessment of *Rpv1*, we also employed SSR markers VVIb32 и VVIM11 mapped in the same chromosome interval as VMC1g3.2 [33]. For the VVIb32 marker, a 154 bp allele was identified in male parents 2000-305-143 and 2000-305-163. The susceptible female parent M. No. 31-77-10 had a different allelic profile (152/152 bp). The same distribution of alleles was observed for the VVIM11 marker: the paternal genotypes 2000-305-143 and 2000-305-163 were heterozygous, carrying the resistant allele of 297 bp, whereas the maternal genotype M. No. 31-77-10 showed an alternative allelic profile (278/284 bp).

In addition to the *Run1/Rpg1* locus on chromosome 12 introgressed from *M. rotundifolia*, other PM resistance loci could be inherited by genotypes 2000-305-143 and 2000-305-163 from *cv*. Regent. As suggested by Zendler et al. [20,21], there are at least two resistant loci to *E. necator* loci on the chromosome 15: *Ren9* (1.1–3.5 cM) and *Ren3* (9.2–9.6 cM), spanning large physical distances between each other. The authors proposed to distinguish between *Ren3* (interval ScORGF15-02—ScORA7*) and *Ren9* (interval CenGen7–GF15-10). The SSR marker GenGen6 closely linked to CenGen7 was suggested for selection of the resistance-mediating haplotype of *Ren9*, while the SCAR marker ScORA7 can be used to detect the resistant allele of *Ren3* inherited by Regent from its parent *cv*. ‘Chambourcin’. We analyzed the parental genotypes of our hybrid populations with the markers developed for the *Ren9* and *Ren3* loci and revealed that male genotypes 200-35-143 and 200-35-163 inherit different haplotypes from Regent in the *Ren9–Ren3* interval assessed by markers GenGen6, UDV116, and ScORA7. The male parent 200-35-143 carries the resistance-associated haplotype identical to the ‘Chambourcin’ grandparent [20], while 200-35-163 has inherited the chromosome fragment from the susceptible predecessor ‘Diana’ (Table 3).

## 3. Discussion

The climate in the south coast of Crimea is close to dry subtropical (Mediterranean) conditions that have favored viticulture on the peninsula since ancient times. Currently, the area of vineyards in Crimea has reached the level of 31,000 ha, with an average yield of 4.2 t ha^−1^, and 82.8% of the vineyard area is occupied by technical grape varieties and 17.2% by table varieties [34]. Fungal diseases have been and remain the main problem for viticulture in this area.

Following the classification of phytopathogens by harmfulness, powdery mildew should be attributed to the main diseases in the western foothill–coastal region of Crimean viticulture, since it causes yield losses from 10% to 50% [35]. Oidium, according to this classification, is definitely the dominant disease, because the yield loss in some years reaches 100%. The development of oidium is epiphytotic almost every year. For example, 8 of 10 years of observation (2004–2014) at the stationary experimental site ‘Livadia’ showed that the development of the disease on bunches of ‘Muscat White’ variety before harvesting was 85.9–100%. Only in 2005 and 2007 did the disease develop moderately (42.8% and 46.5% of bunches were affected, respectively) due to high daytime air temperatures and air drought in the first half of July. Thus, seven to eight sprayings of grapes with highly effective fungicides during the growing season are a common practice in vineyard care [35].

The discovery of the *Run1* gene in American Muscadine grapes (*M. rotundifolia*), immune against PM, opened up new possibilities in viticulture [31]. The BC4 hybrid VRH3082-1-42, derived from the *M. rotundifolia* × *V. vinifera* cross [12], was employed in breeding resistant grapevines using a gene-pyramiding scheme e.g., [31,36]. It was reported that the defense mechanism by which *Run1* confers resistance appears to be different from another dominant gene for PM resistance, *Ren1*, identified in the Central Asian *V. vinifera* variety Kishmish vatkana [31].

In another study, when evaluating F1 progeny derived from the VHR 3082-1-42 × ‘Regent’ cross, it was stated that the effect of the resistance genes originating from ‘Regent’ is higher for downy mildew, while for powdery mildew, the effect of the *Run1* gene is much higher, and it even covers phenotypically the effect of the resistance genes originating from ‘Regent’ [36]. This report is consistent with our results obtained by phytopathological assessment and by the SSR marker assay of two hybrids of this F1 progeny, the genotypes 2000-305-143 and 2000-305-163. Only the latter carries resistant alleles of *Ren3* and *Ren9* genes inherited from Regent, while the *Run1* resistant gene was detected in both of them. However, 2000-305-143 and 2000-305-163 showed no difference in the field resistance to both mildew diseases.

Nevertheless, the incorporation of other sources of resistance to PM into a breeding program makes possible the long-term support of the *Run1* gene and decreases the danger of breakdown of resistance in the event that a new pathogen race appears [10,31]. Thus, about 40% of 102 grape accessions from the international, collaborative project VITISANA showed pyramiding of R-loci against powdery mildew [10].

In this study, we described for the first time, to the best of our knowledge, the genetic and agrobiological diversity of populations of remote hybrids obtained using Magarach 31-77-10 as a female parent by crossing with donors of *Muscadinia rotundifolia* introgressions. The genetic relatedness and distinctiveness of three hybrid populations were assessed using 12,734 SNP markers, generated by Illumina-based genotyping by sequencing (GBS) approach. For all SNP markers, the corresponding position in the reference *V. vinifera* genome was determined and its allelic state in the analyzed grape genotypes was described. The available SNP data set can further be employed as a tool for evaluation of the *M. rotundifolia × V. vinifera* BC populations that are involved in grapevine-breeding programs around the world.

Although our results of the resistance studies and field evaluation of the hybrid genotypes are preliminary (two years of study), they suggest that the three hybrid populations could be a valuable source for breeding new grape varieties well adapted to both abiotic and biotic stresses of the Crimean environment. First, genotypes with hermaphrodite flowers predominate in all three populations: 81% in population 2-11, 52% in population 3-11, and 65% in population 4-11. Thus, the stability of fertilization, which determines the yield, can be less influenced by unfavorable weather conditions.

Additionally, long-term field monitoring of disease resistance against a natural infectious background showed that all hybrid populations demonstrate high resistance to powdery mildew. The infectious pressure is always present in the experimental field where other grapevine varieties grow together with the hybrid populations. Here, for some grape varieties, up to 91% of the spread of oidium on vegetative organs and up to 100% of damage to the generative organs were recorded, while the spread of oidium on vegetative organs of the hybrids, e.g., from the M. No. 31-77-10 × 2000-305-163 cross (population 4-11) never exceeded 20% (Figure 7).

In the present study, we experimentally proved that the progeny of M. No. 31-77-10 × 2000-305-143 and M. No. 31-77-10 × 2000-305-163 crosses are protected from pathogens of powdery mildew and downy mildew by dominant alleles of genes *Run1* and *Rpv1* inherited from their male parents. Evaluation of productivity trait variation among the hybrid populations showed that population 4-11 (♀M. No. 31-77-10 × 2000-305-163) is the most consistent in terms of the strength of plant growth and productivity-related traits (percentage of fruitful shoots, mass of an average bunch, and yield per plant), showing the lowest coefficients of variation. This indicates that the genotypes in this population are quite homogeneous, and most of them are characterized by high productivity. Several progenies of the M. No. 31-77-10 × 2000-305-163 cross with good agrobiological performance were also recognized as promising for winemaking.

In contrast to population 4-11, the hybrids from the M. No. 31-77-10 × [DRX-M5-734, DRX-M5-753, DRX-M5-790] cross are diverse, generally less productive, but some of the hybrid genotypes are characterized by an intense accumulation of sugar.

Among hybrids of population 3-11 (♀M. No. 31-77-10 × 2000-305-143), the outstanding genotype M. No. 3-11-2-41 was discovered, showing the highest score of resistance to powdery mildew, an average mass of bunches (241 g; maximal 662 g), and high average yield (349 g) per shoot (Supplementary Materials, Figure S2).

The current breeding programs at the ‘Magarach’ Institute focus on creation of new varieties with complex immunity to the insect pest *Daktulosphaira vitifoliae* and pathogens *Plasmopara viticola* and *Erysiphe necator* based on the introgression of resistance genes from the highly resistant (immune) species *Muscadinia rotundifolia.* Among hybrids from the crossing of the hybrid genotype Magarach 31-77-10 with the donors of *Muscadinia rotundifolia* genes, progeny have been obtained in which genotypes with high productivity and high resistance to pathogens are distinguished. These are recommended for further agrobiological and technological evaluation as promising candidates for new varieties.

## 4. Materials and Methods

### 4.1. Plant Material and Field Evaluation

Three hybrid populations derived from the cross of female M. No. 31-77-10 (Nimrang × Seibel 13-666) × male 2000-305-143, 2000-305-163, and a mixture of pollen from DRX-M5-734, DRX-M5-753, and DRX-M5-790 were investigated in this study. The crosses were performed in 2011. In 2012, the hybrid seeds were germinated, and in 2013, seedlings were planted as self-rooted plants in experimental fields at the Partenit village, Yalta District, Crimea (44°34′12.0″ N 34°19′44.1″ E), in a self-rooted culture with a planting scheme of 3.5 × 1 m^2^. All the 139 hybrid genotypes were represented by one plant each. The F1 progeny were maintained in an experimental vineyard that was left unsprayed with fungicides.

The area of the field is formed by valleys, ridges, and slopes with different exposure. Soils of the experimental fields are characterized as brown mountainous non-carbonate, with a heavy, loamy gravelly texture. The mild climate provides the possibility of uncovered grape culture: the average annual air temperature is 13.2 °C, the sum of active air temperatures (above 10 °C) is 3700–4055 °C, the number of days with temperature above 10 °C varies between 195 and 218, and the annual precipitation is 300–400 mm. In summer, rains are short, in the form of downpours. Plants often suffer from a lack of moisture because of the dry summer.

Assessment of agrobiological traits was conducted in 2019–2020 according to the method of Lazarevsky [37]. In short, for each genotype, the following traits were recorded: number of latent buds, number of developed shoots, number of fertile shoots, number of inflorescences, number of bunches, average bunch weight (g), and yield per plant (kg). The actual yield from a bush was recorded by counting all bunches on the bush and weighing them.

Phytopathological field evaluation was conducted by the examination of untreated plants against a natural infection pressure. In each season, two counts were carried out: the first in 15–20 days after flowering of grapes and the second at the beginning of grape ripening.

The nature and percentage of leaf damage were scored according to the recommended method [38]. Precisely, on each counting bush, up to 50 leaves were evaluated from both sides for signs of infestations. The percentage of affected leaves and the degree of disease development on each leaf were determined using a scale:

0—no signs of infestation
1—single, hardly visible spots on leaves (OIV resistance—9 points)
2—up to 10% of leaf surface affected (OIV resistance—7 points)
3—11–25% of leaf surface affected (OIV resistance—5 points)
4—26–50% of leaf surface affected (OIV resistance—3 points)
5—more than 50% of leaf surface affected (OIV resistance—1 point)

Using the scale, the disease development score (R, %) in a particular genotype was calculated by the formula

(1) $$R=\frac{\sum a\times b}{N\times K}\times 100$$

where a is a score of the scale, according to which the lesion was evaluated in the experiment; b is the number of affected leaves within the range of this score; N is the total number of leaves evaluated (pcs); K is the highest score of the scale; and 100 is the conversion factor.

For example, of 48 leaves evaluated for a plant, 0 degree of disease development was determined for 27 leaves, 1 for 18 leaves, 2 for 3 leaves, and 3, 4, and 5 for 0 leaves. The disease development score (R, %) was calculated as follows:

(2) $$\frac{\left(0\times 27+1\times 18+2\times 3+3\times 0+4\times 0+5\times 0\right)\times 100\;}{48\times 5}=10\%$$

### 4.2. Genotyping Using Illumina HiSeq2500

#### 4.2.1. DNA Isolation for RADseq Genotyping

Fresh leaves were collected from parental genotypes and hybrid progenies and frozen in liquid nitrogen. DNA was isolated from the plant material using the protocol described by Rahimah et al. [39] with some modifications. Specifically, 160 mg of frozen leaf material was ground with Precellys 24 tissue homogenizer (Bertin Technologies, Montigny le Bretonneux, France) at 2500 rpm for 30 s. To each of the ground samples, 800 µL of modified CTAB buffer (2% CTAB *w*/*v*, 20 mM EDTA (pH 8.0), 1.4 M NaCl, 100 mM Tris-HCl (pH 8.0), 5 mM ascorbic acid, 4mM diethyldithiocarbamic acid sodium salt, and 2% polyvinylpyrrolidone-40), 3.2 µL of 2-mercaptoethanol, and 0.4 µL RNase A were added. The samples were incubated at 37 °C for 30 min, followed by another 30 min at 65 °C for enzyme inactivation. Then, 800 µL of chloroform:isoamyl alcohol (24:1) was added and mixed thoroughly. The mixture was centrifuged at 10,000 rpm for 15 min at room temperature, and the upper aqueous phase was transferred into a sterile 1.5 mL Eppendorf tube. DNA was precipitated by adding 0.7 volume of ice-cold isopropanol. The solution was stored at −80 °C for 15 min and centrifuged at 12,000 rpm at 4 °C for 15 min. The DNA pellet obtained was washed in 200 µL of wash buffer (76% ethanol and 10 mM ammonium acetate) and centrifuged at 14,500 rpm for 5 min. Then, the wash buffer was removed with an FTA-1 Aspirator (BioSan, Riga, Latvia). The pellet was dried at room temperature and suspended in 160 µL of TE buffer (10 mM tris-HCl (pH 8.0) and 1 mM EDTA (pH 8.0)). Extracted DNA was purified by adding to each DNA sample 0.5 volume of 7.5 M ammonium acetate (pH 7.7) and incubating in ice for 20 min. After centrifugation at 12,000 rpm for 15 min at 4 °C, the supernatant was transferred into a clean 1.5 mL Eppendorf tube. The DNA in the supernatant was re-precipitated by adding 2.5 volumes of ethanol, mixed thoroughly, and centrifuged at 12,000 rpm at 4 °C for 15 min. The supernatant was removed, and the DNA pellet was washed with 200 µL of 76% ethanol, dried at room temperature for 15 min, and suspended in 40 µL of TE buffer.

The quality of DNA samples was evaluated with a SPECTROstar Nano spectrophotometer (BMG LABTECH, Ortenberg, Germany), and the optical density was determined at A260, A280, and A350. The integrity of the DNA was further examined by electrophoresing approximately 5 µg of DNA in 1% agarose gel in 1× TAE buffer (0.04 M tris base, 20 mM acetic acid, and 2 mM EDTA). The concentration of each DNA sample was estimated using Qubit 4.0 Fluorometric Quantitation (Thermo Fisher Scientific, Waltham, MA, USA).

#### 4.2.2. Construction and Sequencing of ddRAD Libraries

ddRADseq libraries were prepared, as described previously [21]. ddRAD libraries were constructed from 151 individual DNA samples. The concentration of DNA for each individual sample was normalized to 10 µg/µL, and then 10 µL of the obtained DNA solution was digested for 1 h at 37 °C with 0.7 units of *HindIII* (NEB, Ipswich, MA, USA) in a 20 µL reaction volume. Digestion was stopped by incubating the reaction mixture at 65 °C for 20 min. Next, the barcode adapter was ligated to the end generated by *HindIII*, allowing pooling of the samples. The ligation reaction was carried out at 22 °C for 1 h in a volume of 50 µL with 1.6 units of T4 ligase (NEB, Ipswich, MA, USA). T4 ligase inactivation was reached by heating at 65 °C for 10 min. Next, 10 µL of each sample was pooled and simultaneously purified with AMPure XP beads. The second restriction digestion was performed with 0.7 units of *NlaIII* (NEB, USA) in a 20 µL reaction volume at 37 °C for 1 h with subsequent inactivation at 65 °C for 20 min. The second common adapter was ligated to the overhanging end of *NlaIII*. The reaction mixture (50 µL) was dispersed into 14 aliquots, and PCR was performed on the 14 aliquots to amplify the DNA fragments. The PCR mix (50 µL) contained 1× HF buffer, 25 µL of each primer, 12.5 µM dNTPs, and 1 unit of Phusion^®^ High-Fidelity DNA Polymerase (NEB, USA). PCR conditions were as follows: 98 °C for 30 s, 14 cycles of 98 °C for 10 s, 65 °C for 30 s, 72 °C for 15 s, and then 72 °C for 2 min. All 14 PCR reactions were pooled and purified with AMPure XP beads. The concentration and size distribution of the prepared libraries were checked by automated electrophoresis with the 2100 Bioanalyzer (Agilent Technologies, Santa Clara, CA, USA). Sequencing was performed on Illumina HiSeq2500 with single end reads of 150 base pairs at the CERBALAB Company (St. Petersburg, Russia, https://www.cerbalab.ru/, accessed on 18 December 2020).

#### 4.2.3. SNP Calling

Illumina reads were mapped to the *V. vinifera* 12X genome assembly (https://www.ncbi.nlm.nih.gov/assembly/GCF_000003745.3/, accessed on 18 September 2017) using BWA V. 0.7.17 under default mapping parameters.

The SNP calling procedure was performed using the Tassel V.5.2.40 GBS v2 plug-in [24] and Stacks v.2.53 bioinformatic software [35]. For filtration of raw SNPs, VCFtools v. 0.1.16 [25] was applied, using the following parameters: minor allele frequency more than 1%, maximum missing calls 60%, minimum depth 5, and keeping only biallelic sites. PCA was performed using R v. 4.0.3.

### 4.3. SSR Marker Analysis

For SSR marker analysis, DNA was isolated from 100 mg of fresh young leaves using the CytoSorb kit (Syntol, Moscow Russia) according to the manufacturer’s recommendations.

Amplification reactions were performed in a total volume of 25 µL with 20 ng of template DNA, 10 pmol of each primer, and 10 µL of 2.5× PCR reaction mixture containing 0.25 mM dNTPs, 3 mM MgCl_2_, and 3 units of Taq polymerase with antibodies inhibiting enzyme activity. The forward primer of each pair was 5′-end-labeled with a fluorescent dye (FAM, TAMRA, or R6G) (Syntol, Moscow, Russia).

PCR was carried out using a T100 thermal cycler (Bio-Rad Laboratories, Hercules, CA, USA). The cycling program consisted of the following steps: 5 min at 95 °C, followed by 35 cycles of 15 s at 95 °C, 25 s at 58–66 °C, and 25 s at 72 °C, and a final extension step of 7 min at 72 °C. 

Fragment analysis was performed using a 4-capillary system of the ABI 3130 Genetic Analyzer (Applied Biosystems, Waltham, MA, USA) and Nanofor 05 (Syntol, Russia) using SD-600 labeled with LIZ (Syntol, Russia) as an internal size standard. The PCR fragments were sized with the software GeneMapper (v. 4.0) (Applied Biosystems) and the software DNA Analysis (v. 5.0.3.2.; IAI RAS, Russia).

## Figures and Tables

**Table 1:** Number of SNPs identified for three hybrid populations (2-11, 3-11, and 4-11) using two different SNP callers with and without filtering.

SNP Caller	Number of SNPs Called		
	2-11	3-11	4-11
Stacks	127,178	99,480	86,701
Tassel V5	238,223	137,331	95,187
Stacks filtered	46,085	49,276	41,602
Tassel V5 filtered	24,903	6107	3042

**Table 2:** Variation of the yield-contributing agrobiological traits between flowering plants of the hybrid populations analyzed. Field evaluation data were averaged for 2019 and 2020.

Trait (per Plant)	Population 2-11	Population 3-11	Population 4-11	Difference between Populations (Kruskal Wallis Test, *p*-Value)
Number of latent buds	12.8 ± 0.55	11.7 ± 0.45	10.6 ± 0.73	0.0293
Number of developed shoots	9.7 ± 0.46	7.9 ± 0.38	9.3 ± 0.59	0.0601
Number of fertile shoots	4.8 ± 0.45	3.8 ± 0.34	6.23 ± 0.62	0.0030
Number of inflorescences	5.8 ± 0.62	5.1 ± 0.53	10.3 ± 1.24	0.0005
Number of bunches	5.2 ± 0.49	4.2 ± 0.42	7.8 ± 0.84	0.0013
Bunch weight (g)	94.4 ± 5.24	167.8 ± 14.29	196.6 ± 12.48	0.0000
Yield per plant (kg)	0.47 ± 0.04	0.72 ± 0.11	1.31 ± 0.13	0.0000

**Table 3:** Assessment of parental genotypes of hybrid populations with SSR markers associated with loci of resistance to powdery mildew and downy mildew.

Loci of Resistance	Linked SSR Markers	M. No. 31-77-10		2000-305-143		2000-305-163	
*Run1*	VMC4f3.1	176	178	**192** ^1^	**192**	**192**	**192**
	VMC8g9	176	176	**159**	174	**159**	174
	VMC1g3.2	124	138	**118**	124	**118**	124
*Rpv1*	VVim11	278	284	284	**297**	284	**297**
	VVIb32	152	152	152	**154**	152	**154**
*Ren9*	GenGen6	277	277	277	**287** ^2^	277	277
*Ren3~Ren9*	UDV116	133	143	126	143	126	152
*Ren3*	ScORA7	-	**720**	-	**720**	-	-

^1^ The allele size associated with resistance is shown in bold. ^2^ The resistance-associated haplotype in chromosome 15 (*Ren9-Ren3*) inherited from *cv.* ‘Chambourcin’ via *cv.* Regent is marked by light gray.

## Data Availability

The data presented in this study are available in this article and the Supplementary Materials here.

## Supplementary Materials

The following are available online at https://www.mdpi.com/article/10.3390/plants10061215/s1, Table S1: The list of SNPs used for PCA; Table S2: SNP site distribution; Figure S1: SNP description (number of homozygous, heterozygous, and missing sites per genotype); Figure S2: The hybrid M. No. 3-11-2-41 from population 3-11 (♀M. No. 31-77-10 × 2000-305-143) with outstanding agrobiological characteristics.
