# Supplementary material for: The Assessment of Agrobiological and Disease Resistance Traits of Grapevine Hybrid Populations (Vitis vinifera L. × Muscadinia rotundifolia Michx.) in the Climatic Conditions of Crimea
Source: Plants (Basel). 2021 Jun 15;10(6):1215. doi: 10.3390/plants10061215 (PMC8232157; doi:10.3390/plants10061215)
Supplement: Supplementary file 1 [file plants-10-01215-s001.zip › Supplementary Figure S2.pptx]

## Slide 1
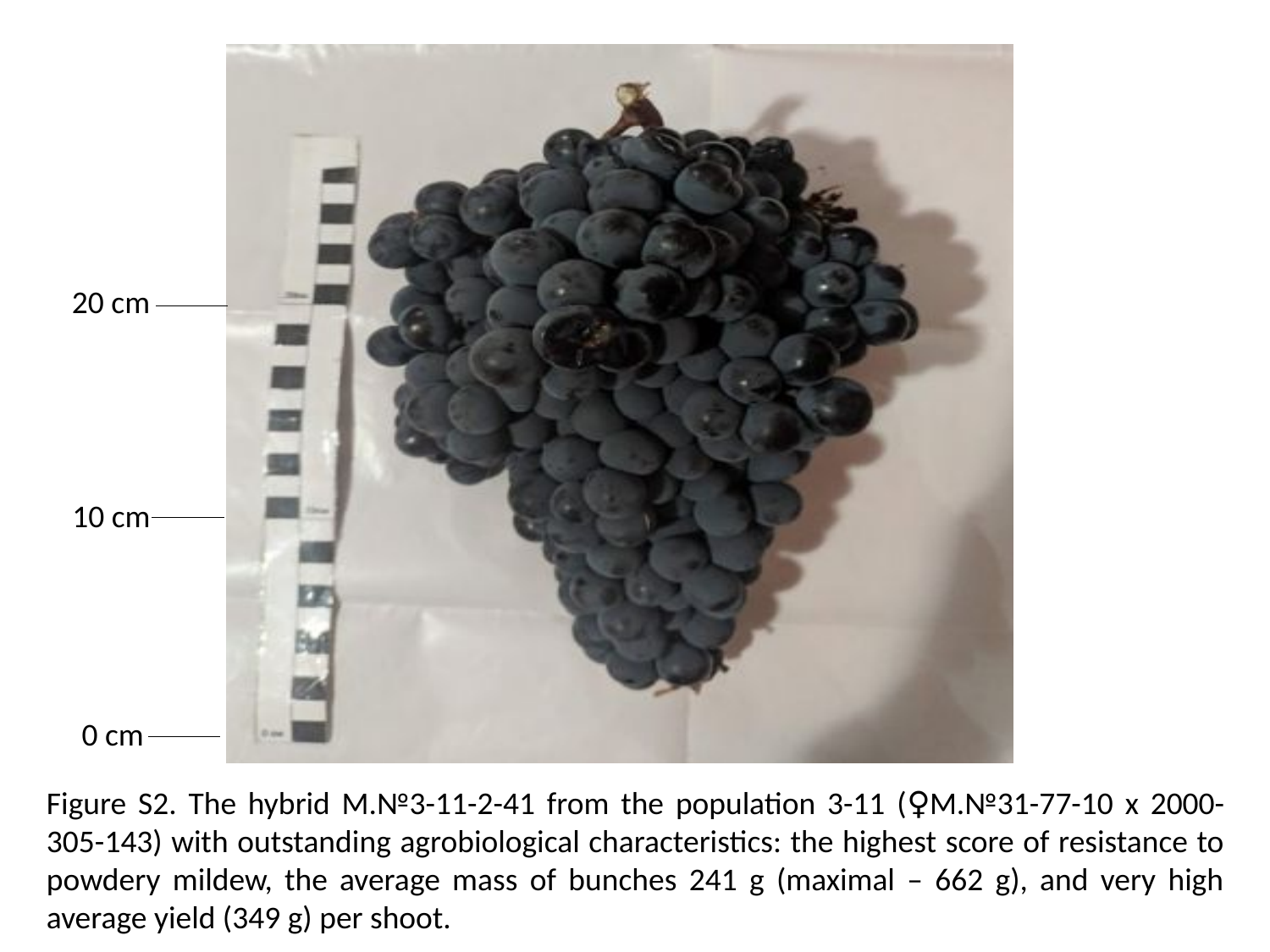

20 cm
10 cm
0 cm
Figure S2. The hybrid М.№3-11-2-41 from the population 3-11 (♀М.№31-77-10 х 2000-305-143) with outstanding agrobiological characteristics: the highest score of resistance to powdery mildew, the average mass of bunches 241 g (maximal – 662 g), and very high average yield (349 g) per shoot.
