# Supplementary figures and images for: The Assessment of Agrobiological and Disease Resistance Traits of Grapevine Hybrid Populations (Vitis vinifera L. × Muscadinia rotundifolia Michx.) in the Climatic Conditions of Crimea
Source: Plants (Basel). 2021 Jun 15;10(6):1215. doi: 10.3390/plants10061215 (PMC8232157; doi:10.3390/plants10061215)

## Slide 1
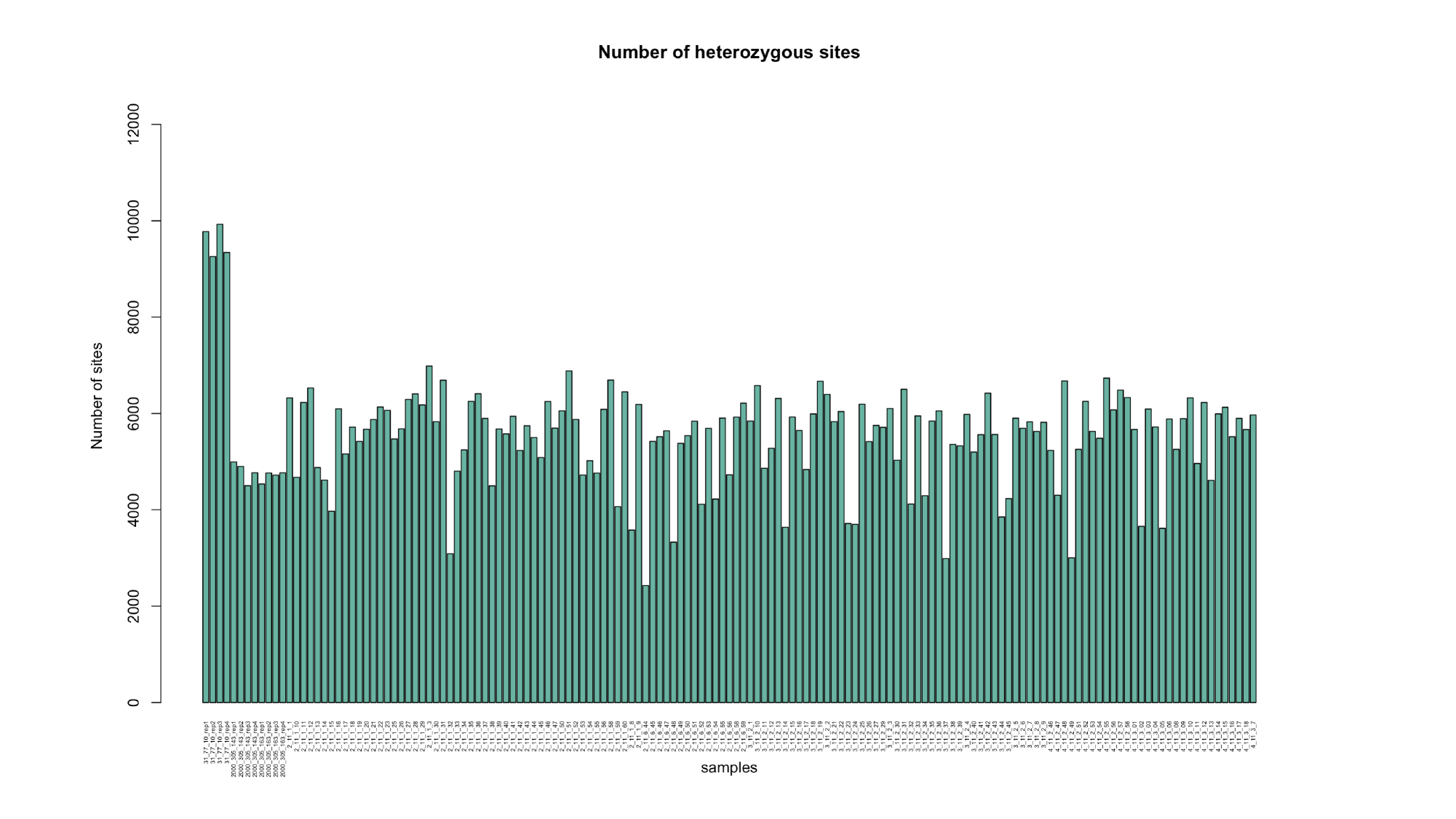

## Slide 2
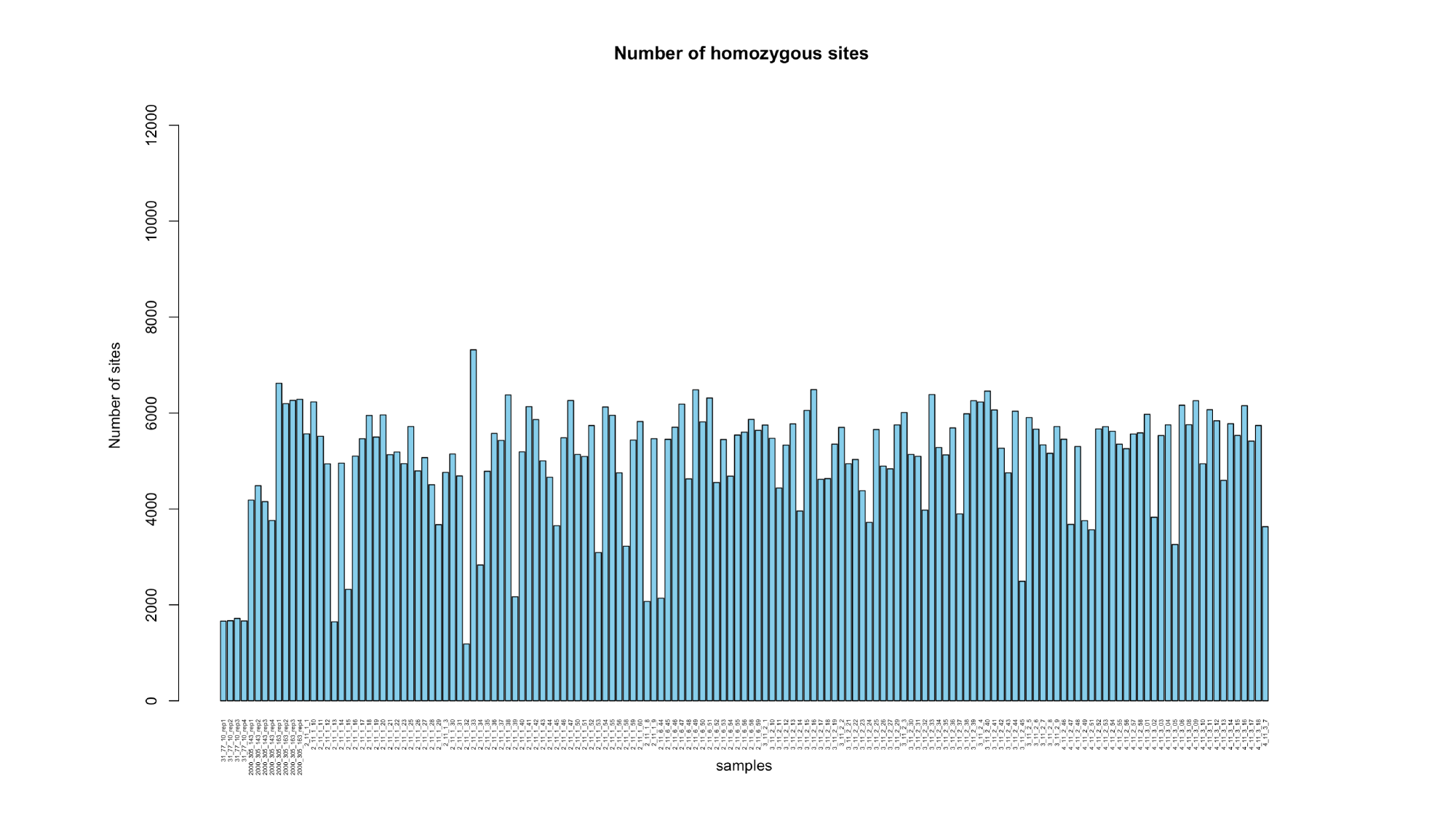

## Slide 3
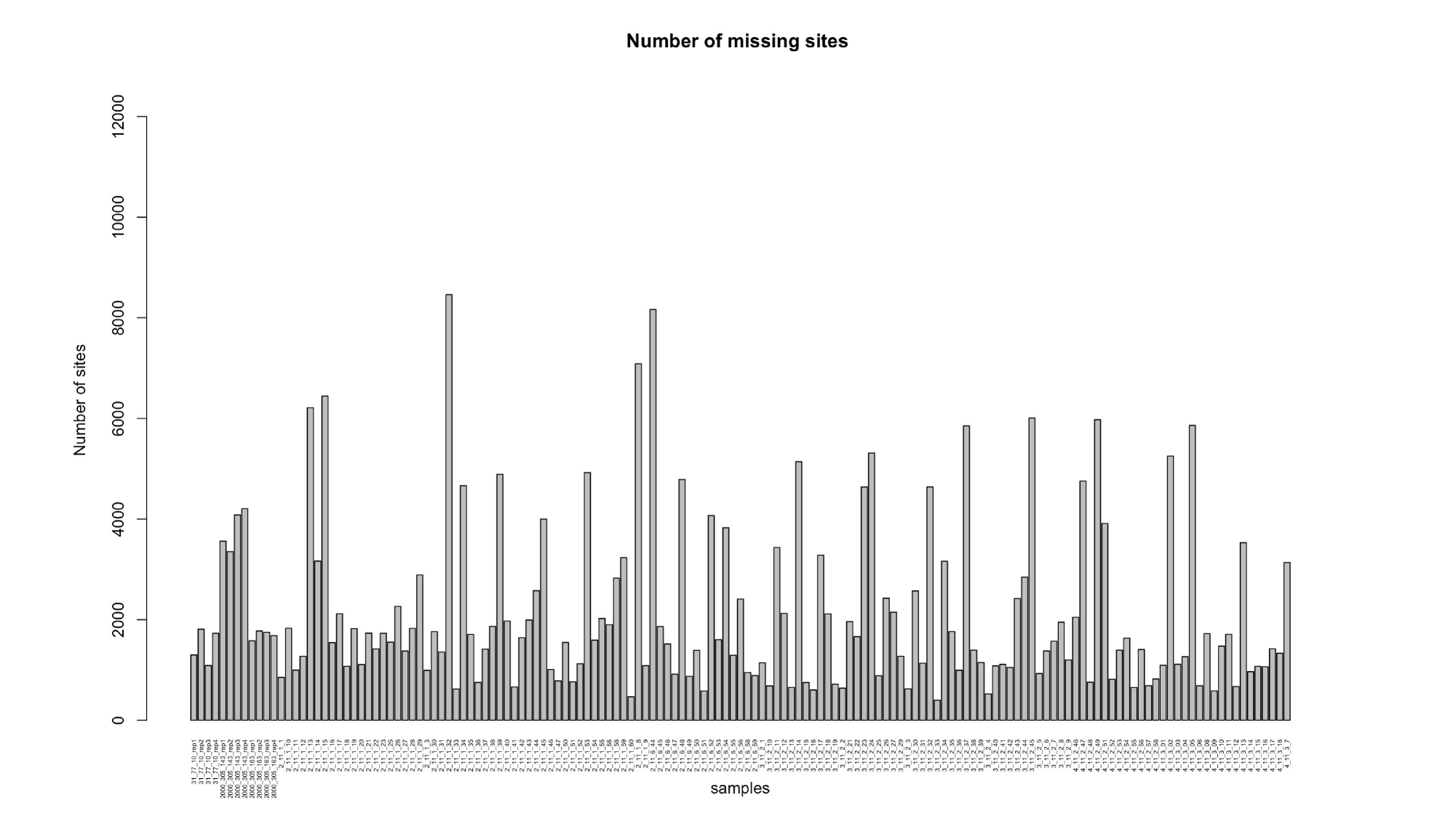

Supplement: Supplementary file 1 [file plants-10-01215-s001.zip › Supplementary Figure S1.pptx]
